# FAMILY MEMBERS’ COMMUNICATION WITH LONG-TERM CARE PROVIDERS AND ITS INFLUENCE ON RESIDENT WELL-BEING

**DOI:** 10.1093/geroni/igac059.2567

**Published:** 2022-12-20

**Authors:** Francesca Falzarano, Verena Cimarolli, Karen Siedlecki

**Affiliations:** Weill Cornell Medicine, Douglaston, New York, United States; LeadingAge, Washington, District of Columbia, United States; Fordham University, Bronx, New York, United States

## Abstract

Considerable research has examined communication dynamics among family members and staff in nursing homes (NHs) and has demonstrated that better communication is associated with more optimal psychosocial outcomes in both family caregivers and formal care providers. However, the literature on how communication dynamics influence resident functioning is limited, and it has yet to be determined how communication impacts residents across other care contexts, such as Assisted Living Facilities (ALFs). Thus, using data from the National Health and Aging Trends Study and the National Study on Caregiving, the purpose of this study was to examine family perceptions of communication with formal care providers (i.e., frequency, availability, and helpfulness of communication) and its influence on resident outcomes in two samples of long-term care residents (n=337 in ALFs, n=112 in NHs) and their family caregivers, and to compare how results differ across care setting. When examining the full sample of long-term care residents, findings showed that better communication was associated with lower depressive symptoms and negative affect. When investigating differences across care settings, we found that those residing in NHs exhibited higher levels of depressive and anxiety symptoms compared to ALF residents. Further, better communication was associated with lower levels of depressive symptoms only among ALF residents. Our findings provide insights into how interpersonal dynamics between family and formal care providers influence resident functioning and underscores the importance of enhanced communication among all members of the primary care team – that is, healthcare providers, residents, and their family members.

